# Comparative functional analyses of PHR1, PHL1, and PHL4 transcription factors in regulating Arabidopsis responses to phosphate starvation

**DOI:** 10.3389/fpls.2024.1379562

**Published:** 2024-04-19

**Authors:** Zhen Wang, Zai Zheng, Dong Liu

**Affiliations:** ^1^Faculty of Agriculture, Forestry and Medicine, The Open University of China, Beijing, China; ^2^Ministry of Education Key Laboratory of Bioinformatics, Center for Plant Biology, School of Life Sciences, Tsinghua University, Beijing, China; ^3^Hainan Yazhou Bay Seed Laboratory, Sanya, Hainan, China

**Keywords:** PHR1, PHL1, PHL4, Pi starvation responses, flowering time, transcriptomic analyses, functional relationship

## Abstract

To cope with phosphate (Pi) starvation, plants trigger an array of adaptive responses to sustain their growth and development. These responses are largely controlled at transcriptional levels. In Arabidopsis (*Arabidopsis thaliana*), PHOSPHATE RESPONSE 1 (PHR1) is a key regulator of plant physiological and transcriptional responses to Pi starvation. PHR1 belongs to a MYB-CC-type transcription factor family which contains 15 members. In this PHR1 family, PHR1/PHR1-like 1(PHL1) and PHL2/PHL3 form two distinct modules in regulating plant development and transcriptional responses to Pi starvation. PHL4 is the most closely related member to PHR1. Previously, using the *phr1phl4* mutant, we showed that PHL4 is also involved in regulating plant Pi responses. However, the precise roles of PHL1 and PHL4 in regulating plant Pi responses and their functional relationships with PHR1 have not been clearly defined. In this work, we further used the *phl1phl4* and *phr1phl1phl4* mutants to perform comparative phenotypic and transcriptomic analyses with *phr1*, *phr1phl1*, and *phr1phl4.* The results showed that both PHL1 and PHL4 act redundantly and equally with PHR1 to regulate leaf senescence, Pi starvation induced-inhibition of primary root growth, and accumulation of anthocyanins in shoots. Unlike PHR1 and PHL1, however, the role of PHL4 in maintaining Pi homeostasis is negligible. In regulating transcriptional responses to Pi starvation at genomic levels, both PHL1 and PHL4 play minor roles when acts alone, however, they act synergistically with PHR1. In regulating Pi starvation-responsive genes, PHL4 also function less than PHL1 in terms of the number of the genes it regulates and the magnitude of gene transcription it affects. Furthermore, no synergistic interaction was found between PHL1 and PHL4 in regulating plant response to Pi starvation. Therefore, our results clarified the roles of PHL1 and PHL4 in regulating plant responses to Pi starvation. In addition, this work revealed a new function of these three transcription factors in regulating flowering time.

## 1 Introduction

Phosphorus (P) is an essential macronutrient for plant growth, development, and metabolism. Although P is abundant in the environment, its major form for plant uptake, phosphate (Pi), is quite limited in soils due to its low diffusion rate, fixation with metals, and conversion to organophosphates by microorganisms (Nussaume et al., 2011; Cong et al., 2020). To cope with low Pi stress, plants have evolved a series of developmental and metabolic responses called Pi starvation responses (PSR) to sustain their growth, development, and reproduction. The typical PSR include the inhibition of primary root (PR) growth, the increase of lateral roots and root hairs, the enhancement of activity of high-affinity Pi transporters on the root surface, the secretion of acid phosphatases, and the accumulation of anthocyanins and starches in leaves (López-Arredondo et al., 2014). Accompanying these adaptive responses is a dramatic genome-wide reprograming of gene transcription (Wu et al., 2003; Misson et al., 2005; Morcuende et al., 2007; Bustos et al., 2010; Osorio et al., 2019; Hani et al., 2021).

PHOSPHATE RESPONSE 1 (PHR1) was first identified in the model plant Arabidopsis (*Arabidopsis thaliana*) as a central regulator of plant transcriptional responses to Pi starvation (Rubio et al., 2001). With its MYB domain, PHR1 binds to a *cis*-element named P1BS (PHR1-binding sequence) with an imperfect palindromic sequence of 5’-GNATATNC-3’. This *cis*-element occurs in the promoters of many Pi starvation-induced (PSI) genes (Bustos et al., 2010). Knock-out of *PHR1* greatly reduces the transcription of PSI genes and affects several Pi starvation responses, such as inhibition of PR growth, accumulation of anthocyanins in shoots, secretion of acid phosphatases on the root surface, and maintenance of Pi homeostasis (Rubio et al., 2001; Sun et al., 2016; Wang et al., 2018). In contrast, overexpression of *PHR1* enhances the expression of PSI genes and consequently increases the cellular Pi content in plants irrespective of Pi regimes (Nilsson et al., 2007). The transcriptional activity of PHR1 is regulated by an SPX domain-containing protein, SPX1. Under Pi sufficiency, the level of cellular inositol pyrophosphate InsP_8_ is high. The high level of InsP_8_ bring SPX1 and PHR1 together to form a complex that prevents PHR1 from binding to the P1BS element on the promoters of PSI genes; under Pi deficiency, the level of InsP_8_ is decreased, resulting in the release of PHR1 from the complex. The released PHR1 then binds to the promoters of PSI genes and increase their transcription (Wild et al., 2016; Dong et al., 2019; Ried et al., 2021). PHR1 also acts as a hub in the interactions between Pi and other essential nutrients, such as nitrate (Hu and Chu, 2020), sulfate (Rouached et al., 2011), zinc (Khan et al., 2014), iron (Bournier et al., 2013), and calcium (Linn et al., 2017), as well as phytohormone jasmonic acid (Khan et al., 2016; He et al., 2023). The functional orthologs of PHR1 have been identified in other plant species, including soybean (Xue et al., 2017), oilseed rape (Ren et al., 2012), rice (Zhou et al., 2008), and wheat (Wang et al., 2013), suggesting PHR1 is an evolutionarily conserved and important regulator of plant transcriptional responses to Pi starvation.

**Figure 1:**
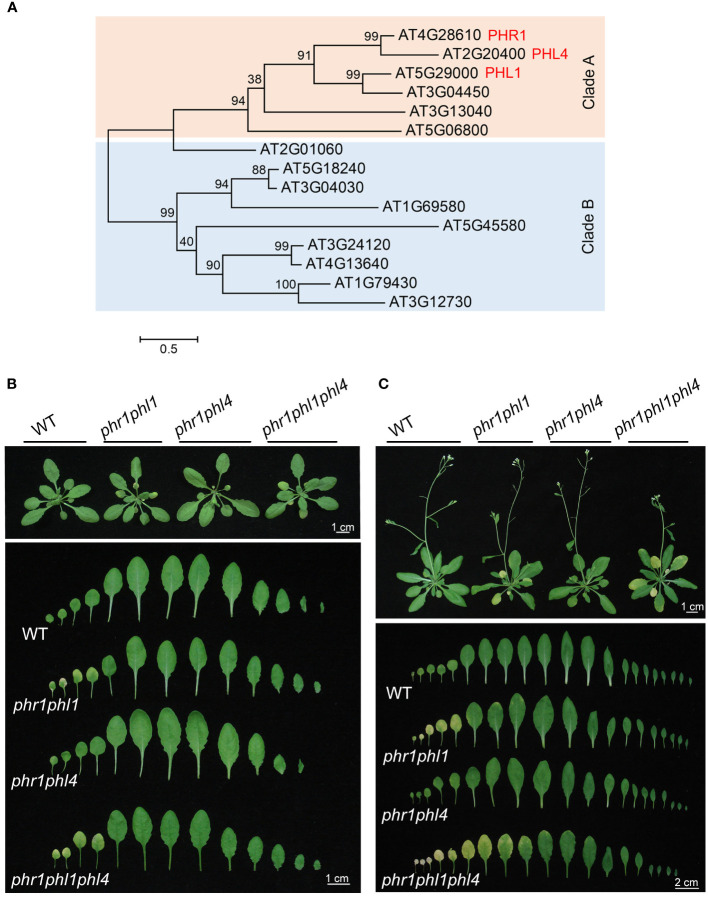
The phylogenetic relationship of *Arabidopsis* PHR1 family members and growth phenotypes of the WT and various mutants. **(A)** The phylogenetic tree of the PHR1 family members generated by MEGA 7 software. Multiple alignment was conducted by an online service, Clustal Omega (https://www.ebi.ac.uk/Tools/msa/clustalo/). A maximum likelihood (ML) algorithm and full-length protein sequences were used to construct the tree. **(B)** and **(C)** Up panels: Growth phenotypes of one-month **(B)** and 45-day-old **(C)** plants of the WT, *phr1phl1*, *phr1phl4*, and *phr1phl1phl4* grow in soil. The senescence phenotype is indicated as yellowish leaves. Bottom panels: A close view of the rosette leaves that were arranged from the oldest to the youngest (from left to right).

In Arabidopsis, PHR1 belongs to a MYB-CC protein family which contains 15 members (Rubio et al., 2001, **Figure 1A**). These 15 members are divided into two clades and PHR1 is in Clade A. PHR1-LIKE1 (PHL1) in Clade A is a close relative of PHR1. The mutation of *PHL1* has little effect on the expression of PSI genes. In *phr1phl1* double mutant, however, the expression of PSI genes is greatly enhanced compared to that in *phr1*, indicating that PHR1 and PHL1 form a functional module to synergistically regulate PSI gene expression (Bustos et al., 2010). Distinct from PHR1 and PHL1, PHL2 and PHL3 within the Clade B together acts as another module to regulate plant transcriptional responses to Pi starvation (**Figure 1A**, Sun et al., 2016; Wang et al., 2023).

In PHR1 family, PHL4 is located in Clade A and is most closely related to PHR1 (**Figure 1A**). Previously, we reported that PHL4 also functions redundantly with PHR1 in regulating PSI gene expression by comparative analyses of *phr1*, *phr1phl1*, and *phr1phl4* (Wang et al., 2018). However, whether synergistic interactions also exist between PHL1 and PHL4 and how the triple mutation of *phr1phl1phl4* would affect plant development and PSI gene transcription at genomic levels has not been investigated. In this work, we further generated the *phl1phl4* and *phr1phl1phl4* mutants to conduct comparative phenotypic and transcriptomic studies with *phr1* and *phr1phl1* to determine the functional relationships among PHR1, PHL1, and PHL4. Our results further support the previous conclusion that PHL1 and PHL4 along only play minor role in regulating plant transcriptional responses to Pi starvation and need to act with PHR1 to exert a strong effect; and between these two proteins, PHL4 functions less even though it is most related to PHR1.

## 2 Materials and methods

### 2.1 Plant materials and growth conditions

All Arabidopsis (*Arabidopsis thaliana*) plants used in this study were in the Colombia-0 (Col-0) ecotype background. The T-DNA insertional lines SALK_067629 (*phr1*) and SAIL_731_B09 (*phl1*) were obtained from the Arabidopsis Biological Resource Center (ABRC). These two mutants have been proved to be the null mutants (Nilsson et al., 2007; Bustos et al., 2010). The null allele of *PHL4* gene, *phl4-2* (referred as *phl4* in this work), was generated by CRISPR/Cas9-based genome editing system (Wang et al., 2018). *phr1phl1*, *phr1phl4*, *phl1phl4*, and *phr1phl1phl4* were produced through genetic crossing using above mentioned single mutants. *PHL4 OX-1* (referred as *PHL4 OX* in this work) was generated as described in Wang et al., 2018.

Surface-sterilized seeds were sown on Pi-sufficient (+Pi) or Pi-deficient (-Pi) medium. The +Pi medium contained half-strength Murashige & Skoog basal salts (Caisson Labs, MSP01-01190008), half-strength Murashige & Skoog vitamin powder (1000×) (Phyto Technology Laboratories, M533), 1.0% (w/v) sucrose, 0.5% MES, and 1.2% (w/v) agar (Sigma-Aldrich, catalog no. A1296) or 0.8% (w/v) agarose (Biowest, catalog no.111860). The pH of the medium was adjusted to 5.8 with NaOH. In the -Pi medium, half-strength Murashige & Skoog without Pi (Caisson Labs, MSP11-05160009) was used to replace Murashige & Skoog basal salts. After seeds were stratified for 2 days at 4°C, the agar plates were placed vertically in a phytotron with a photoperiod of 16 h light and 8 h dark at 22-24°C. The light intensity was 100 μmol m^-2^ s^-1^. *Nicotiana benthamiana* plants were grown in soil under the same lighting conditions.

### 2.2 Quantification of cellular Pi content and total P content

Cellular Pi content and total P content were quantified according to Wang et al. (2011). Specifically, the weighed fresh shoot and root tissues were submerged in 1 ml of 1% glacial acetate and were alternatively freeze-thawed 10 times in liquid nitrogen and in a water bath maintained at 65°C. A 100-μl volume of the extract was mixed with 200 μl of ddH_2_O and 700 μl of Pi reaction buffer containing a mixture of [0.48% NH_4_MoO_4_, 2.85% (v/v) H_2_SO_4_] and [10% (w/v) ascorbic acid] in a ratio of 6:1. The reaction was allowed to proceed at 37°C for 1 h. The cellular Pi content was determined at A_820_ according to a prepared standard curve and was expressed as μmol/g FW.

To determine the total P content, about 50 mg of fresh tissues was oven-dried at 500°C for 3 h and flamed to ash. The ashes were dissolved in 100 μl of 30% (v/v) HCl and 10% (v/v) HNO3. About 10 μl of the dissolved sample was mixed with 290 μl of ddH_2_O and 700 µl of Pi reaction buffer, and the Pi was quantified by above method. The total P contents of plant tissues were determined and expressed as Pi contents extracted from flamed ashes.

### 2.3 Quantification of anthocyanin content

Anthocyanins in shoots were extracted with propanol: HCl: H_2_O (18: 1: 81, v/v/v) in dark at room temperature for 24 h. Absorbance was measured at 530 and 650 nm. Anthocyanin content was expressed as (A_530_ – A_650_)/g FW.

### 2.4 Reverse transcription quantitative PCR analyses of PSI gene expression

Total RNAs of 8-day-old seedlings were extracted using the Magen HiPure Plant RNA Mini Kit. A 2 μg quantity of the RNAs were reversely transcribed to cDNA using M-MLV reverse transcriptase (Takara). qRT-PCR analyses were carried out using 2×RealStar Green Fast Mixture (GenStar, catalog no. A301-10) on a Bio-Rad CFX96 real-time PCR detection system. The *ACTIN 2* gene (At3g18780) was used as an internal control, and the relative expression level of each gene was calculated by the 2^−ΔΔCt^ method (Livak and Schmittgen, 2001). The primers used for RT-qPCR analyses are listed in **Supplementary Table 1**.

### 2.5 Luciferase complementation imaging and bimolecular fluorescence complementation assays

For LCI assays, the CDSs of *PHR1*, *PHL1*, and *PHL4* were inserted into the vectors pCAMBIA-nLUC and pCAMBIA-cLUC (Chen et al., 2008), respectively. For BiFC assay, the CDSs of *PHR1*, *PHL1*, and *PHL4* were individually cloned into the vector nYFP or cYFP using a one-step isothermal *in vitro* recombination procedure (Gibson et al., 2009). The resultant constructs were mobilized into the *Agrobacterium tumefaciens* strain GV3101 and used to transform the leaves of *Nicotiana benthamiana*. The LCI and BiFC assays were performed as described by Sun et al. (2016). The primers used for vector construction are listed in **Supplementary Table 1**.

### 2.6 Yeast two-hybrid assays

The yeast two-hybrid assays were performed using the Matchmaker GAL4 Two-Hybrid System (Clontech) according to the manufacturer’s instructions. The CDSs of *PHR1* and *PHL1* were cloned into pGBKT7 vector, while the CDS of *PHL4* was cloned into pGADT7 vector. The various combinations of the two constructs were co-transformed into the yeast strain AH109. Transformants were selected on SD/-Trp/-Leu/-His medium and SD/-Trp/-Leu/-His/-Ade medium.

### 2.7 Transcriptomic analysis

The RNAs of the root of 8-day-old Col, *phr1*, *phl1*, *phl4*, *phr1phl1*, *phr1phl4*, *phr1phl1phl4*, and *PHL4 OX* grown on +Pi and –Pi medium were extracted and sent to Anoroad genome company for RNA-sequencing analysis. Three biological replicates were used in this experiment. Sequencing was performed on an Illumina platform generating 150bp pair-end reads. The expression levels of genes were normalized by TPM (transcripts per million). Genes with a fold change of |Log_2_FC|≥1 and FDR<0.05 were considered as differentially expressed. Comparisons were made between -Pi and +Pi wild-type plants to identify PSR genes (**Supplementary Table 2**) as well as Pi-deficient mutant and wild-type plants to identify mutant-affected genes (**Supplementary Table 4**). Heatmap was drawn using Morpheus (https://software.broadinstitute.org/morpheus/). GO enrichment was performed using the Agrigo v2 (http://systemsbiology.cau.edu.cn/agriGOv2/).

## 3 Results

### 3.1 PHL1 and PHL4 act with PHR1 to regulate leaf senescence and flowering time

To determine the functional relationship among PHR1, PHL1, and PHL4 in regulating plant development and responses to Pi starvation, we further generated Arabidopsis *phl1phl4* double mutant and *phr1phl1phl4* triple mutant through genetic cross (for details, see Materials and Methods). We first compared their growth phenotypes with *phr1*, *phl1*, *phl4*, *phr1phl1*, and *phr1phl4* in soils. The seeds of the WT and all the mutants were germinated on 1/2 MS medium and grown for seven days before they were transferred to soils. Previously, we reported that none of the single mutant of *phr1*, *phl1*, or *phl4* showed obvious developmental defects in soils, but *phr1phl1* showed early senescence phenotype (Wang et al., 2018, 2023). This work also showed that the one-month-old soil-grown plants of *phr1phl1* and *phr1phl1phl4*, but not of *phr1phl4*, displayed early senescence phenotype (yellowish leaves) (**Figure 1B**). When the plants became 45-days old, *phr1phl1phl4* had more senescent leaves and higher degree of senescence than those of *phr1phl1* (**Figure 1C**). *phr1phl1phl4* also flowered later than the WT and *phr1phl1*. These results suggested that PHL1 and PHL4 not only act redundantly with PHR1 in regulating leaf senescence, but also in regulating flowering time.

### 3.2 PHL1 and PHL4 play equally minor roles in regulating Pi starvation-induced inhibition of PR growth and accumulation of anthocyanins

**Figure 2:**
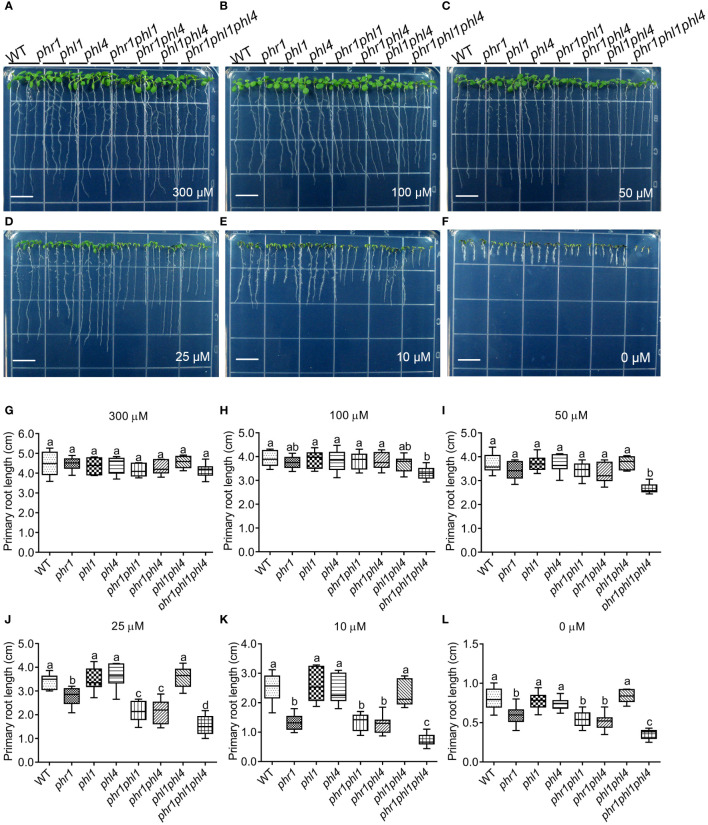
PR growth of 8-day-old WT and various mutants grown on media with different amount of Pi supplementations. **(A–F)** The pictures showing the morphologies of the seedlings grown on the media with 300 μM, 100 μM, 50 μM, 25 μM, 10 μM, or 0 μM Pi supplementations, respectively. The Pi content in the medium is indicated at the bottom of each panel. Bars = 1 cm. **(G–L)** Quantification of PR length of the seedlings grown in different media. The Pi supplementation in the medium is indicated on the top of the panel. These experiments were repeated three times with similar results. Values represent means ± SD of more than ten primary roots for each genotype. Different letters above any two columns within the same chart indicate significant differences of the values between these two samples (One-way ANOVA/Tukey test, *P* < 0.05).

Next, we analyzed developmental and physiological responses of the mutants to Pi starvation. The seeds of the WT and all the mutants were directly germinated on 1/2 MS media with different amount of Pi supplementations and primary root (PR) growth was evaluated eight days after germination (8 DAG). When the Pi concentration was reduced from 625 μM Pi (normal 1/2 MS medium, or +Pi medium) to 300 μM, the seedlings of all the mutants displayed similar PR length with that of the WT (**Figures 2A, G**). When Pi concentration was reduced to 50 μM, the PR length of all the mutants still did not differ from that of the WT, except *phr1phl1phl4* whose PR length was only about 70% of the WT (**Figures 2C, I**). When grown on the medium with 25 μM Pi, the PR length of *phr1*, but not *phl1* and *phl4*, was shorter than the WT (**Figures 2D, J**). On this medium, the PR growth of two double mutants and the triple mutant also became progressively more inhibited than *phr1*. On the medium with 10 μM Pi or without Pi supplementation (-Pi medium), the PR lengths of *phr1*, *phr1phl1*, and *phr1phl4* were similar, but that of *phr1phl1phl4* was further reduced (**Figures 2E, F, K, L**).

Induction of anthocyanin production in shoots is a hallmark response of plants to Pi starvation. We then analyzed the anthocyanin contents in the shoots of these mutants. The seeds of plants with different genotypes were sown on +Pi and -Pi media and grown for 12 days with strong light intensity (200 μmol m^-2^ s^-1^) before the analyses were performed. On +Pi medium, all types of seedlings accumulated very low levels of anthocyanins in their shoots (**Supplementary Figure 1**). Under -Pi condition, WT seedlings accumulated a large quantity of anthocyanins in their shoots. The anthocyanin content of *phr1*, but not of *phl1* and *phl4*, was reduced to about 20% of the WT. Both *phr1phl1* and *phr1phl4* displayed further reduction of anthocyanin content with similar extents compared with *phr1*. The triple mutant *phr1phl1phl4* did not show further decrease of anthocyanin content compared with *phr1phl1* (**Supplementary Figure 1**).

The above results together indicated that PHR1 plays a major role, and PHL1 and PHL4 play equally minor role in regulating Pi starvation-induced inhibition of PR growth and accumulation of anthocyanins.

### 3.3 PHL4 plays weaker role than PHL1 in regulating Pi homeostasis

**Figure 3:**
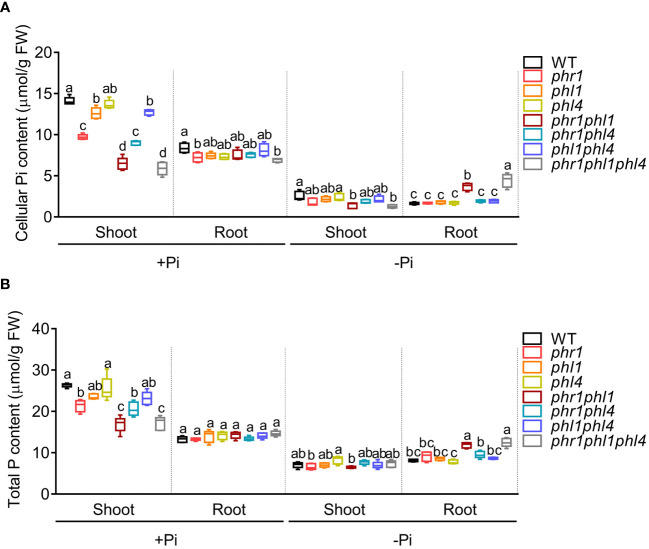
The cellular Pi content **(A)** and total P content **(B)** of 10-day-old WT and mutants grown on +Pi and –Pi media. These experiments were repeated three times with similar results. Values represent means ± SD of more than five replicates. Different letters above any two columns within the same chart indicate significant differences of the values between these two samples (One-way ANOVA/Tukey test, *P* < 0.05).

Finally, we investigated the role of PHR1, PHL1, and PHL4 in regulating Pi homeostasis by analyzing the Pi contents in all mutants at 10 DAG (**Figure 3A**). Under +Pi condition, the Pi content of *phr1* decreased to about 60% of the WT in shoots. The Pi content of *phr1phl1*, but not *phr1phl4* displayed further decrease compared to that of *phr1*. *phl1* and *phl1phl4* also had a small but significant reduction of Pi content compared to that of the WT. In +Pi shoots, *phr1phl1phl4* did not show further decrease of Pi content compared to *phr1phl1*. In Pi sufficient-roots and Pi deficient-shoots, Pi contents of all mutants were similar to that of the WT. However, in Pi-deficient roots, the Pi content of *phr1phl1* was almost doubled of that of the WT, consistent with what we reported before (Wang et al., 2018, 2023). The Pi content of *phr1phl1phl4* were further increased compared to that of *phr1phl1* (**Figure 3A**). We also measured the total P contents in all mutants. The total P contents in all samples were closely correlated with their cellular Pi levels (**Figure 3B**). These results suggested that PHR1, PHL1, and PHL4 redundantly regulated Pi homeostasis, but both PHL1 and PHL4 only played a minor role. And, among them, PHL4 had the least contribution.

### 3.4 PHL4 has a stronger interaction with PHL1 than with PHR1

**Figure 4:**
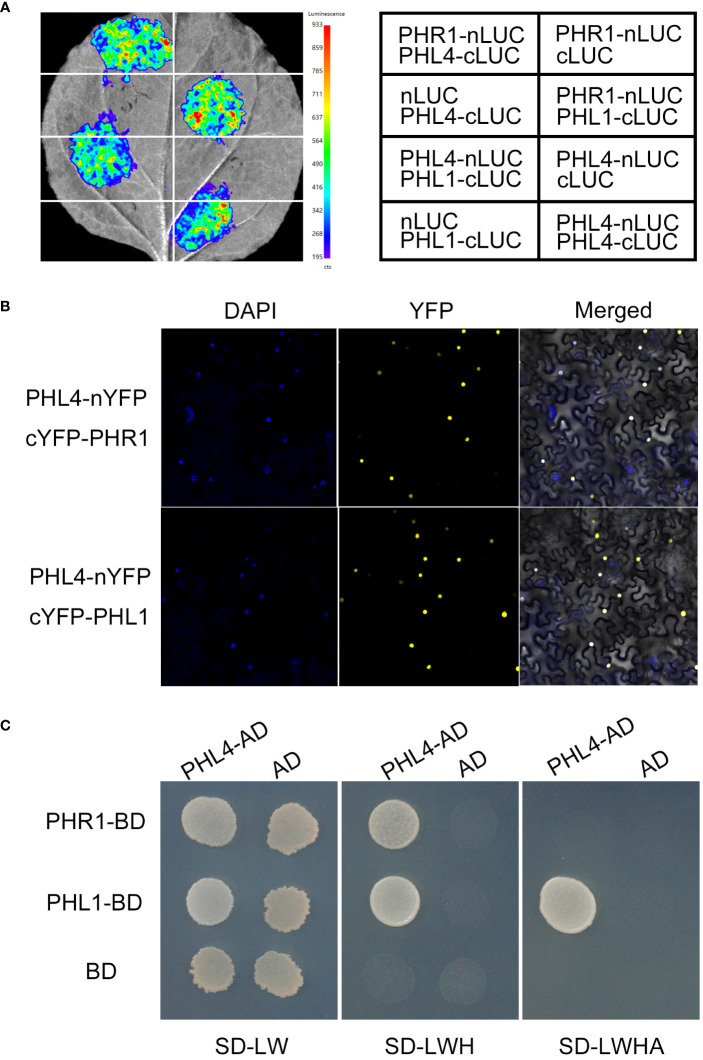
Physical interactions among PHR1, PHL1, and PHL4. **(A)** The interactions among PHR1, PHL1, and PHL4 tested by luciferase complementation imaging assays. **(B)** The interactions among PHR1, PHL1, and PHL4 tested by bimolecular fluorescence complementation assays. **(C)** The interactions among PHR1, PHL1, and PHL4 tested by yeast-two hybrid experiments. From left to right: the yeast cells carrying different combination of the construct grown on the double-, triple-, and quadruple-deficient selection media.

Previously, PHR1 has been shown to directly interact with PHL1 and PHL4 (Bustos et al., 2010; Wang et al., 2023). To determine whether PHL4 could also directly interact with PHL1, we first performed luciferase (LUC) complementation imaging assays. The coding sequences (CDS) of *PHL1* and *PHL4* were fused to the C-terminal half of the luciferase (cLUC) and the CDS of *PHR1* was fused to the N-terminal half of the LUC (nLUC). *PHL1-cLUC* and *PHL4-cLUC* were co-transformed with *PHR1-nLUC*, respectively, into the leaves of *N. benthamiana* with proper controls (*nLUC* or *cLUC*). All the gene constructs mentioned in this study were under the control of *35S CaMV* promoter. Like *PHL1-cLUC* and *PHR1-nLUC*, the co-expression of *PHL4-cLUC* and *PHR1-nLUC* resulted in a strong fluorescence signal from reconstituted LUC activity (**Figure 4A**), consistent with the previous reports (Bustos et al., 2010; Wang et al., 2023). A *PHL4-nLUC* construct was also made. When this *PHL4-nLUC* fusion gene was co-expressed with *PHL1-cLUC* or *PHL4-cLUC* in the leaves of *N. benthamiana*, they also generated strong reconstituted LUC signals. These results demonstrated that PHL4 not only could interact with PHL1 but also with itself. The bimolecular fluorescence complementation assays performed in the leaves of *N. benthamiana* supported above conclusions (**Figure 4B**).

We further carried out the yeast two-hybrid experiments to determine the relative strength of the interactions among these transcription factors. The CDS of *PHR1* and *PHL1* were individually fused to the DNA-binding domain of the transcription factor GAL4 (PHR1-BD and PHL1-BD) while the CDS of PHL4 was fused to its transcriptional activation domain (PHL4-AD). The yeast cells carrying *PHR1-BD* and *PHL4-AD* or *PHL1-BD* and *PHL4-BD* constructs were grown on the selection media. The results not only confirmed the interactions between PHL4 and PHL1, and PHL4 and PHR1, but also indicated that the interaction between PHL4 and PHL1 was stronger than that between PHL4 and PHR1 (**Figure 4C**).

### 3.5 Global view of the transcriptomic changes induced by Pi starvation of the WT and mutants

To determine the individual roles of PHR1, PHL1, and PHL4 in regulating plant transcriptional responses to Pi starvation at genomic levels, we conducted transcriptomic analyses of the roots of WT, *phr1*, *phl1*, *phl4*, *phr1phl1*, *phr1phl4*, *phr1phl1phl4* and *PHL4*-overexpressing lines. Total RNAs were extracted from the roots of 8-day-old seedlings grown under +Pi and -Pi conditions. We first performed RT-qPCR analyses of six hallmark PSI genes for all batches of RNAs. These six PSI genes included a non-coding transcript, *IPS1* (Burleigh and Harrison, 1999); a microRNA, *miR399D* (Fujii et al., 2005); two high-affinity phosphate transporters, *AtPT1* (*Pht1;1*) and *AtPT2* (*Phtl;4*) (Muchhal et al., 1996); a ribonuclease, RNS1 (Bariola et al., 1994); and an acid phosphatase, *ACP5* (*AtPAP17*) (del Pozo et al., 1999). The results showed that the expression of all six genes were significantly induced in the WT (**Supplementary Figure 2**), indicating that the experimental condition was set up properly. These batches of RNAs were then subjected to sequencing using next generation sequencing technology. Three biological replicates were used for all genotypes. The RT-qPCR analyses of the same six PSI genes for all the mutants were also closely correlated with the results from RNA-seq data, validating the approaches of RNA-seq experiments (**Supplementary Figure 2**).

Using |Log_2_FC| ≥ 1 (differential expression level greater than 2 folds) and FDR (false discovery rate) < 0.05 as cutoff, we identified 2579 PSR genes which included 1339 PSI genes and 1240 Pi starvation-suppressed (PSS) genes for the WT (**Supplementary Table 2**). In this work, we focused our analyses only on the PSI genes for all genotypes. GO analysis showed that most PSI genes identified in the WT were involved in glucosinolate biosynthetic process (GO:0019761), Pi transport (GO:0006817), cellular response to Pi starvation (GO:0016036) (**Supplementary Table 2**). The remaining PSI genes were involved in responses to abiotic and biotic stresses such as hypoxia, wounding, salt, water deprivation, pathogens as well as in some hormone signaling pathways, including jasmonic acid, karrikin, abscisic acid. These results were largely consistent with what we reported recently (Wang et al., 2023) and with those reported by other research groups in the past (Wu et al., 2003; Misson et al., 2005; Morcuende et al., 2007; Bustos et al., 2010; Osorio et al., 2019; Hani et al., 2021).

**Figure 5:**
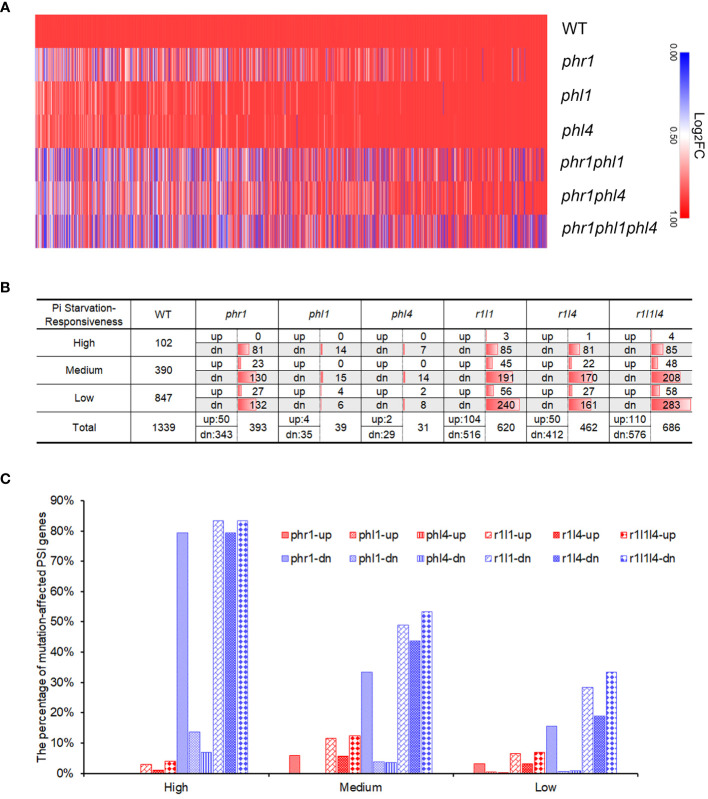
Summary of the differentially expressed genes in the WT and various mutants. **(A)** Heatmap showing the expression levels of the PSI genes in the WT and various mutants. The value (Log_2_FC) for each PSI gene in the heatmap was calculated by comparing the expression of Pi-deficient plants of each genotype to the expression of the Pi-sufficient WT. The diagram was generated using an online software, Morpheus (https://software.broadinstitute.org/morpheus/). **(B)** The numbers of PSR genes with different responsiveness affected in each mutant. **(C)** The percentages of PSR genes with different responsiveness affected in each mutant. In **(B, C)**: PSI genes were divided into three categories according to their responsiveness to Pi starvation: “High” refers to Log_2_FC > 5, “Medium” refers to 2 < Log_2_FC ≤ 5, and “Low” refers to 1 ≤ Log_2_FC ≤ 2. “up” refers to the PSI genes whose expression is upregulated in the mutants; “dn” refers to the PSI genes whose expression is downregulated in the mutants.

To obtain a global view of the Pi starvation-induced changes of transcriptomes for the WT and all the mutants, we drew a heatmap of PSI genes using the Log_2_FC value of Pi-deficient WT and Pi-deficient mutants *versus* Pi-sufficient WT. Our visual examination revealed that under Pi deficiency, the transcriptome of *phr1* displayed significant changes compared to that of the WT, whereas those of *phl1*and *phl4* only showed a slight change (**Figure 5A**; **Supplementary Table 3**). Both *phr1phl1* and *phr1phl4* double mutants showed further alterations of the transcriptomes compared to that of *phr1*, but the alterations in *phr1phl4* was lower than those in *phr1phl1. phr1phl1phl4* showed the further changes of the expression profile compared to those of two double mutants. The heatmap therefore indicates that PHR1, PHL1, and PHL4 all were involved in regulating PSI gene expression, but PHL1 and PHL4 alone only played minor roles, and between these two, PHL4 contributed the least.

### 3.6 The distinct roles of PHR1, PHL1, and PHL4 in regulating transcriptional responses to Pi starvation

Next, we investigated the individual roles of PHR1, PHL1, and PHL4 in regulating the expression of PSI genes by quantitative analyses. To determine how the mutation of these three PHR family members affected gene expression under Pi starvation, we defined those genes in the mutants whose expression was significantly higher than those in the WT as “PSI-up genes” and those genes whose expression was significantly lower than those in the WT as “PSI-down genes”. We also arbitrarily used a 2-folds difference of expression levels between the WT and a mutant with FDR < 0.05 to define a given PSI gene as “significantly mutation-affected”. With these criteria, we found that the expression of the majority of mutation-affected genes was downregulated compared to those in the WT (**Figure 5B**; **Supplementary Table 4**). This result indicated that the major functions of PHR1, PHL1, and PHL4 were the promotion of gene transcription under Pi starvation. In *phr1*, the expression of total 393 PSI genes was affected; among them, 343 were downregulated and 50 were upregulated; whereas in *phl1* and *phl4*, only 39 and 31 PSI genes whose expression were affected, respectively, i.e. 35 downregulated and 4 upregulated in *phl1* and 29 downregulated and 2 upregulated in *phl4* (**Figure 5B**). The number of mutation-affected genes was increased in two double mutants, but the increase in *phr1phl1* (620) was larger than the increase in *phr1phl4* (462). The number of mutation-affected genes in *phr1phl1phl4* was further increased to 686. These results demonstrated there were synergistic interactions among PHR1, PHL1, and PHL4 in regulating the transcription of PSI genes.

All the PSI genes were further divided into three categories according to their responsiveness to Pi starvation (High: Log_2_FC > 5; Medium:2 < Log_2_FC ≤ 5; Low: 1 ≤ Log_2_FC ≤ 2). Our analyses indicated that 79.4% of the PSI genes of high Pi responsiveness (Log_2_FC > 5) were downregulated in *phr1* and this percentage decreased to 13.7% in *phl1* and 6.9% in *phl4*, respectively (**Figures 5B, C**). In the double and triple mutants, this percentage did not significantly change compared to that of *phr1* (83.3% in *phr1phl1*, 79.4% in *phr1phl4* and 83.3% in *phr1phl1phl4*). In terms of medium-responsive PSI genes, the percentages of PSI-down genes were decreased to 33% in *phr1*, 3.8% in *phl1*, and 3.6% in *phl4*. This percentage was significantly increased to 49.0% in *phr1phl1*, 43.6% in *phr1phl4*, and 53.3% in *phr1phl1phl4*. For the low-responsive PSI genes, the percentages for the PSI-down was further decreased to 15.6% in *phr1*, 0.7% in *phl1*, and 0.9% in *phl4*, respectively. Compared to the single mutants, this percentage was increased to 28.3 in *phr1phl1*, 19.0% in *phr1phl4*, and 33.4% in *phr1phl1phl4*. Taken together, these results suggested that when combined with PHR1, PHL1 and PHL4 mutations did not significantly increased the number of high-responsive PSI-down genes, but increased the number of medium- and low-responsive PSI-down genes. Moreover, compared to PHL1, PHL4 has weaker enhancing effect when it acted with PHR1.

**Figure 6:**
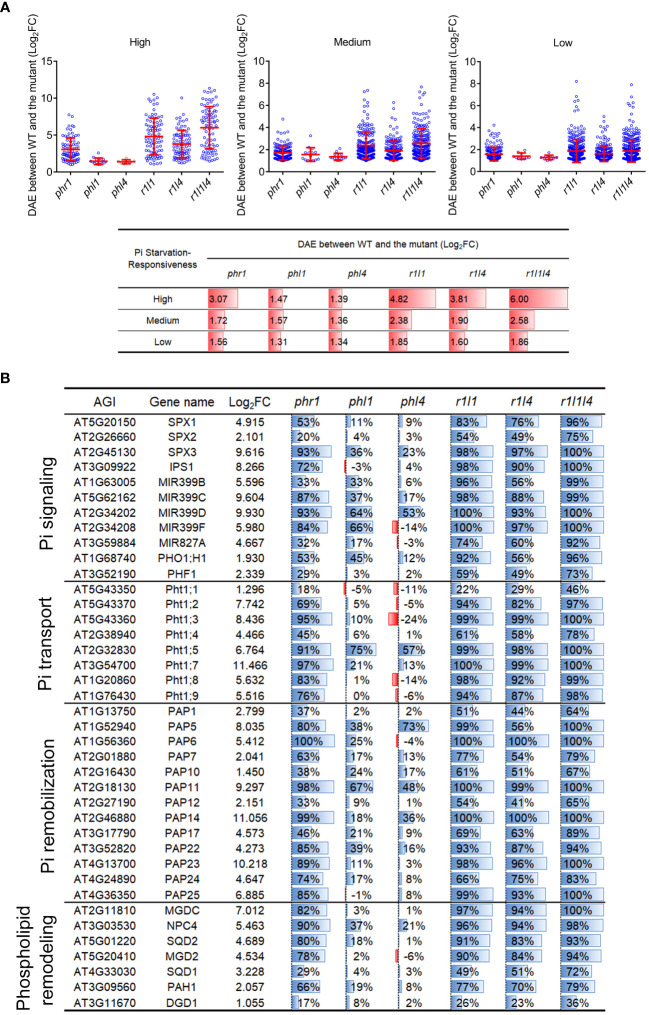
The difference of average expression levels (DAE) of mutation-affected PSI-down genes and the expression levels of some typical PSI genes in the mutants. **(A)** The DAE of mutation-affected PSI-down genes between the WT and each mutant. Up panel: The scatter plots showing means ± SD of DAE for each mutant. The mutant names are indicated in X-coordinate. Bottom panel: the corresponding data showing in up panel. All PSI-down genes examined were divided into three categories according to their responsiveness to Pi starvation: “High” refers to Log_2_FC > 5, “Medium” refers to 2 < Log_2_FC ≤ 5, “Low” refers to 1 ≤ Log_2_FC ≤ 2. **(B)** The expression of typical PSI genes in mutants. The third column indicates the Log_2_FC between Pi-sufficient and Pi-deficient WT. The fourth to ninth column shows the percentages of decreased transcription level in each mutant compared to the WT.

Considering that the mutation of *PHL1* and *PHL4* in *phr1* background did not significantly increase the number of mutation-affected high-responsive PSI-down genes, we wondered whether the combined mutations might more affect the magnitude of PSI gene transcription. We then quantified the difference of average expression level (DAE) of PSI-down genes between the WT and each mutant. For a given mutant, its DAE was determined by calculating the difference of the expression level of each differentially expressed gene between the -Pi WT and -Pi mutant, then taking the average value of these differences (expressed as Log_2_FC) (**Figure 6A**). The DAE of PSI-down genes for *phr1* was highest among all single mutants in all type of PSI-down genes, especially for high-responsive ones. The DAE was increased in both *phr1phl1* and *phr1phl4*, but the increase in *phr1phl1* was more significant than that in *phr1phl4*. Compared to *phr1phl1*, the DAE was further significantly increased in *phr1phl1phl4* for high-responsive PSI-down genes (6.00 *vs* 4.82), but less significantly increased for medium-responsive PSI-down gens (2.58 *vs* 2.38), and not significantly increased for low-responsive PSI-down genes (1.86 *vs* 1.85). When such analyses were performed using all PSI genes, including both PSI-up and PSI-down genes, the results displayed the similar trends (**Supplementary Figure 3**). This was probably because the PSI-up genes only account for a small portion in all the mutants.

Furthermore, we selected a group of PSI genes that are known to be involved in various Pi starvation responses, such as Pi signaling (SPX proteins, IPS1, miRNA399s), Pi transport (Pi transporters), Pi remobilization (purple acid phosphatases, PAP), and lipid remodeling (the enzymes involved in the generation of glycolipids and sulfate lipids to replace phospholipids in biomembranes). The transcriptomic changes of these genes were compared among the WT and each mutant under Pi starvation. For the nine members of *Pht1* family (high affinity Pi transporters, hereafter, were collectively referred as *Pht1* genes), we analyzed all of them except for *Pht1:6* that is specifically expressed in young floral bud and pollen (Nussaume et al., 2011). Mutation of *PHR1* resulted in more than 50% decrease in all *Pht1* genes except for *Pht1;1* (18%) and *Pht1;4* (45%), whereas mutation of *PHL1* and *PHL4* only reduced the expression of *Pht1;5* to less than 50% of the WT (**Figure 6B**). For the double mutants, both *phr1phl1* and *phr1phl4* had further reduction of the expression of *Pht1* genes compared with *phr1*. Overall, the extent of the reduction of *Pht1* gene expression in *phr1phl1* was greater than that in *phr1phl4*. For the triple mutant, the expression levels of the *Pht1* genes were further decreased; in which the expression of six of *Pht1* genes was reduced to less than 5% of that of the WT. The regulatory effects of PHR1, PHL1, and PHL4 on the expression of the PSI genes that were involved in Pi signaling, Pi remobilization, and lipid remodeling were similar with that on *Pht1* genes (**Figure 6B**).

### 3.7 Further evidence for the function of PHL4 in regulating plant transcriptional responses to Pi starvation

**Figure 7:**
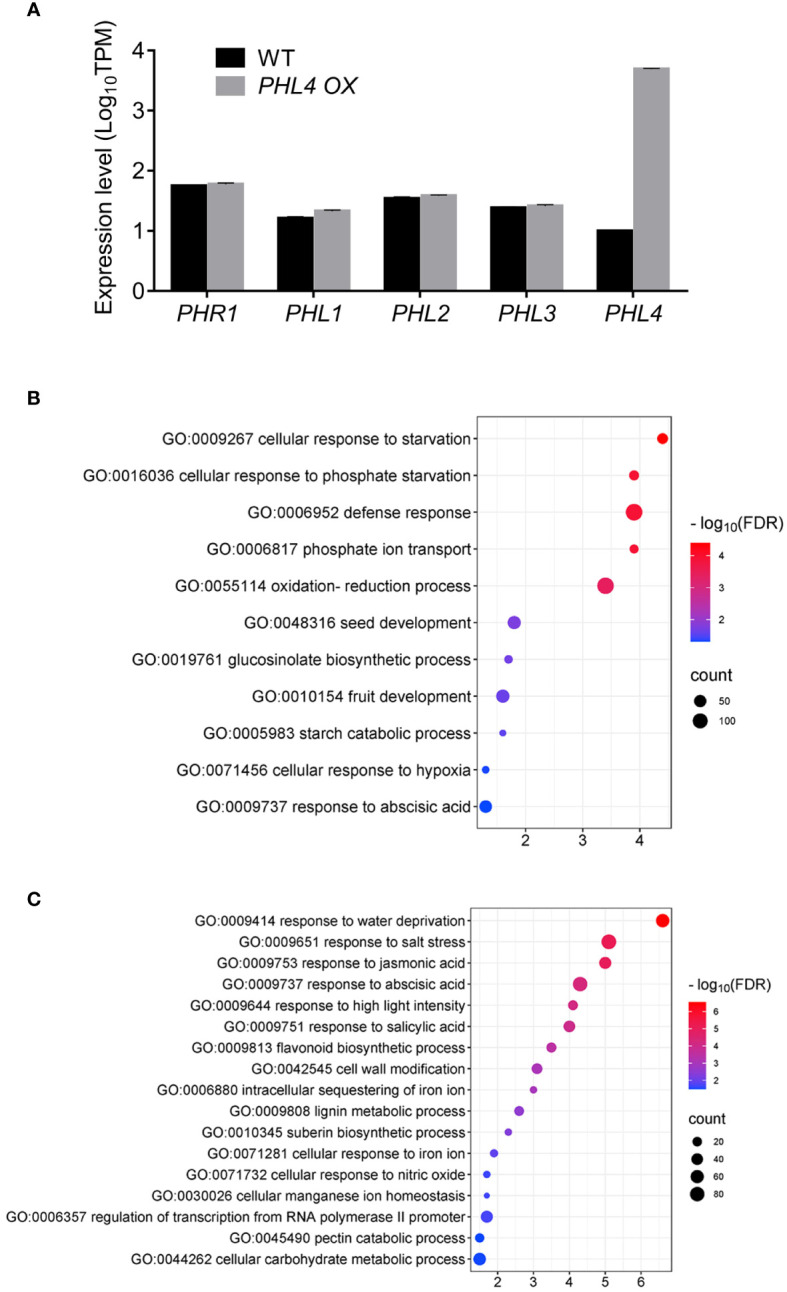
Further evidence for the function of PHL4. **(A)** The expression level of *PHR* genes in *PHL4 OX* line under +Pi condition. Values represent means ± SD of three replicates of the genes in mutants. **(B, C)** The GO enrichments of significantly upregulated **(B)** and downregulated **(C)** genes in *PHL4 OX* line under +Pi condition.

We have previously reported that overexpression of *PHL4* increased plant Pi starvation responses including enhanced root-associated APase activity, elevated cellular Pi and total P contents, and increased expression of typical PSI genes (Wang et al., 2018). To further understand the working mechanism of PHL4, we compared the expression profile between the WT and *PHL4 OX* line by RNA-seq analysis under normal growth condition. Compared to the WT, there were 869 genes whose expression was upregulated and 1213 genes whose expression was downregulated in the *PHL4 OX* line (**Supplementary Table 5**). We then examined whether *PHL4* functions by affecting the expression of other PHR genes. The results showed that in the *PHL4*-overexpressing (*PHL4 OX*) line, the expression of *PHL4* was increased around 500 folds while the expression of other *PHR* genes were not obviously altered (**Figure 7A**).

Finally, we conducted GO analysis of differentially expressed genes at genomic scale in the *PHL4 OX* line under normal growth condition. For those *PHL4 OX*-upregulated genes, they were enriched in cellular responses to Pi starvation (GO:0016036), Pi transport (GO:0006817), including all *Pht1* genes, except for *Pht1:6*, and glucosinolate biosynthetic process (0019761) (**Figure 7B**). For the *PHL4 OX*-downregulated genes, they were enriched in response to abiotic stress (water deprivation, salt stress), hormone-related process (jasmonic acid, abscisic acid, salicylic acid), cell wall-related process (cell wall modification, lignin metabolic process, pectin catabolic process), and Fe-related process (intracellular sequestering of iron ion, cellular response to iron ion) (**Figure 7C**). These *PHL4 OX*-affected genes were largely consistent with the PSR genes observed in Pi-deficient plants (Wang et al., 2023). Therefore, these results provided further evidence for the function of PHL4 in regulating transcriptional responses to Pi starvation.

## 4 Discussion

In Arabidopsis, the central role of PHR1 in controlling plant transcriptional responses to Pi starvation has been well established. In PHR1 family, PHL1 and PHL4 are two closest relatives of PHR1 (**Figure 1A**). The single mutation of *PHL1* or *PHL4* along has no obvious effects on plant development and transcriptional responses to Pi starvation (Bustos et al., 2010; Wang et al., 2018). The *phr1phl1* and *phr1phl4* double mutants, however, exhibit enhanced inhibition of PR growth and more reduced accumulation of anthocyanins under Pi deficiency compared to *phr1* (Wang et al., 2018). Furthermore, *phr1phl1*, but not *phr1phl4*, displays early senescence under normal growth condition and has more profound effects on the expression of six typical PSI genes examined compared to *phr1* (Wang et al., 2018). Although the above results have indicated that both PHL1 and PHL4 are involved in regulating plant responses to Pi starvation, there are still some important questions remained to be answered. For example, if there exists a synergistic interaction between PHL1 and PHL4 like that between PHR1 and PHL1? And, whether the mutation of *PHL4* in the *phr1phl1* background would further greatly enhance the mutant phenotypes?

In this work, the generation of the *phl1phl4* and *phr1phl1phl4* mutants allowed us to determine the precise roles of PHL1 and PHL4 in regulating plant development and responses to Pi starvation. By comparing the phenotypes of WT, *phr1phl1*, *phr1phl4*, and *phr1phl1phl4* under normal growth condition, we found that PHL4 is also involved in regulating leaf senescence, but its contribution is less than PHL1 because its effect on leaf senescence is only shown in *phr1phl1phl4* but not in *phr1phl4* (**Figures 1B, C**). Besides, the late flowering phenotype observed for *phr1phl1phl4*, for the first time, revealed a new function for these three transcription factors in regulating flowering time (**Figure 1C**). This might not be surprising because we previously had reported that *phl2phl3* also has late flowering phenotype (Wang et al., 2023). However, that the late flowering phenotype was not observed for *phr1phl1* and *phr1phl4* suggested that PHR1, PHL1, and PHL4 each only plays a minor role in this developmental process. It would be interesting to combine the mutations of all these five PHR1 family proteins to see how it would affect flowering time and how they interact with those known flowering genes.

In terms of plant developmental and physiological responses to Pi starvation, we first examined the PR growth of all the mutants under five different Pi conditions. By comparing the PR growth of all mutants in five different Pi regimes, we found that *phr1*, but not *phl1* and *phl4*, had more inhibited PR growth than WT under Pi deficiency; and *phr1phl1* and *phr1phl4* showed similar further reduction of PR growth compared to *phr1* (**Figure 2**). These results are consistent with what we previously reported (Wang et al., 2018). *phr1phl1phl4* exhibited more severe inhibition of PR growth than the double mutants; however, PR growth of *phl1phl4* under Pi deficiency did not differ from the WT, suggesting that there was no synergistic interaction between PHL1 and PHL4 in regulating PR growth under Pi starvation. Together, we concluded that PHR1 plays a major role, and PHL1 and PHL4 play equal minor roles in regulating PR growth under Pi deficiency. The same conclusion could also be applied to the functions of these three PHR1 family proteins in the control of Pi deficiency-induced anthocyanin accumulation in shoots.

Under normal growth condition, there was about 40% reduction of Pi contents in the shoots of *phr1* (**Figure 3**). *phl1*, but not *phl4*, also has a very small but significant decrease of Pi content in shoots. When *phl4* was combined with *phr1*, *phl1*, or *phr1phl1*, no further reduction of Pi content was observed in these multiple gene mutants, indicating that the contribution of PHL4 to the control of shoot Pi homeostasis under Pi sufficiency is negligible. These results indicated that PHL4 functions less than PHL1 in regulating Pi homeostasis. Interestingly, under Pi deficiency, the Pi content of *phr1phl1* was almost doubled of that of the WT. And, the Pi content of *phr1phl1phl4* were further increased compared to that of *phr1phl1*. PHO1 is a Pi exporter which plays a key role in translocating Pi from root to shoot (Hamburger et al., 2002; Arpat et al., 2012). We speculated that the increase of Pi contents in the *phr1phl1* and *phr1phl1phl4* might be due to the impaired expression, protein accumulation or transport activity of PHO1 occurred in these mutants. We examined transcriptomic data in *phr1phl1* and *phr1phl1phl4* and found that the expression of PHO1 was not significantly altered (**Supplementary Table 4**). Whether the protein accumulation and transport activity are reduced in these mutants need further investigation.

Previously, we reported that like PHL1, mutation of *PHL4* alone has very minor effects on the expression of six typical PSI marker genes (Wang et al., 2018). Both *phr1phl1* and *phr1phl4* have enhanced reduction of PSI gene expression compared to *phr1*; however, the degree of enhancement is higher for *phr1phl1* than for *phr1phl4*, suggesting PHL4 functions less than PHL1 in regulating PSI gene expression. However, this inference has not been tested at genomic levels. In this work, the RNA-seq experiments showed that the percentage of mutation-affected genes is 29.3% in *phr1*, 2.9% in *phl1* and 2.3% in *phl4*. In the double and triple mutant, this percentage became 46.3% in *phr1phl1*, 34.5% in *phr1phl4*, and 51.2% in *phr1phl1phl4*. These results further supported that in regulating PSI gene expression, PHR1 plays a major role and both PHL1 and PHL4 play minor roles.

The great increase of the number of the mutation affected-PSI genes in *phr1phl1* and *phr1phl4* compared to that in *phr1* indicated that, like that between PHR1 and PHL1, there also exist synergistic interactions between PHR1 and PHL4. However, there was no further large increase in the number of mutation-affected PSI genes in *phr1phl1phl4* compared to *phr1phl1* (**Figure 5**). These results suggested that PHL1 and PHL4 have overlapped targets when individually acts with PHR1. When the PSI genes were further classified into three categories based on the degree of their responsiveness to Pi starvation, we could see that PHR1 regulates 79.4% of high-responsive PSI genes, 33.3% of medium-responsive genes, and 15.5% of low-responsive genes. For PHL1 and PHL4, the percentages of the PSI genes they regulate in these three categories show similar patterns as PHR1, i.e. progressively decrease in high- medium-, low-responsive PSI genes, but the total number of the genes that PHL1 and PHL4 regulated in all three categories is much lower than those PHR1 regulated. In addition, the magnitude of the expression of PSI genes that is affected in *phr1* is much higher than that is affected in *phl1* and *phl4* as indicated by the DAE between the WT and each mutant (**Figure 6**). All these results concluded that as a single protein, PHR1 plays a major role, and PHL1 and PHL4 play minor roles in regulating PSI gene transcription. Furthermore, by comparing both the number of PSI genes and the magnitude of PSI gene expression that affected in *phr1phl1* and *phr1phl4* (**Figures 5**, **6**), we could clearly see that PHL4 functions less than PHL1 in regulating PSI gene expression at genomic level. The less influence of PHL4 on the transcription of the PSI gene which have been known to be involved in Pi starvation responses (**Figure 6B**) could well explain why the mutation of *PHL4* does not have an obvious effect on Pi homeostasis. The functional discrepancies between PHL1 and PHL4 in regulating plant development and Pi transcriptional responses might lie within the different N-terminal sequences of these two proteins, which may affect the degree of their interactions with PHR1. In other words, even though the protein sequence of PHL4 is more related to PHR1 than that of PHL1, the interaction of PHL1 with PHR1 might be stronger than PHL4. The second possibility is that PHL1 might have higher binding affinity to the P1BS element within the promoters of the PSI genes than PHL4. Considering the interactions between PHR1 and PHL1 or PHL4, some important unanswered questions are whether the protein accumulation, transcriptional activity or ability to bind downstream target genes of PHR1 are affected by PHL1 and PHL4 and in what degree they are affected, respectively. In future, we would use various methods to test these hypotheses.

Because even in the *phr1phl1phl4* triple mutant, there was only 51.2% of the total PSI genes whose expression was affected, it meant that there are many PSI genes that are regulated by the transcription factors other than PHR1, PHL1, and PHL4. This is consistent with our previous report that PHL2/PHL3 module also functions in regulating PSI gene expression, especially in regulating medium- and low-responsive PSI genes (Wang et al., 2023). The incomplete coverage of the regulation of all PSI genes indicates that there must be other important regulators inside or outside PHR1 family which need to be identified.

## 5 Conclusion

This work clarified the relative contributions of PHL1 and PHL4 in regulating plant development and responses to Pi starvation. They both played minor and slightly different roles compared to PHR1 when act alone or act with PHR1; and between them, PHL4 functions less than PHL1. Unlike that between PHL1 and PHR1 or between PHL4 and PHR1, there is no synergistic functional interaction between PHL1 and PHL4 although there is a stronger protein-protein interaction between PHL4 and PHL1 than that between PHL4 and PHR1. It would be interesting to investigate why PHL4 functions less than PHL1 even though its protein sequence is most related to PHR1.

## Data availability statement

Publicly available datasets were analyzed in this study. This data can be found here: [http://ncbi.nlm.nih.gov/sra./ accession no. GSE217158].

## Author contributions

ZW: Conceptualization, Data curation, Formal analysis, Investigation, Validation, Writing – original draft, Writing – review & editing, Methodology, Resources, Software, Visualization. ZZ: Data curation, Formal analysis, Investigation, Methodology, Validation, Writing – review & editing. DL: Conceptualization, Data curation, Formal analysis, Funding acquisition, Investigation, Project administration, Supervision, Validation, Writing – original draft, Writing – review & editing.

## Supplementary material

Supplementary Figure 1 The anthocyanin contents of 12-day-old WT and mutants on +Pi and –Pi media.

Supplementary Figure 2 Relative expression of six PSI genes in root of the WT and various mutants.

Supplementary Figure 3 The difference of average expression levels (DAE) of mutation-affected PSI genes (PSI-up and PSI-down genes included) between WT and mutants.

Supplementary Table 1 Primer sequences used in this study.

Supplementary Table 2 Classification of PSR genes.

Supplementary Table 3 Expression values of the PSI genes used to generate the heatmap in **Figure 5A**.

Supplementary Table 4 Mutation-affected PSI genes.

Supplementary Table 5 The *PHL4 OX*-affected genes under Pi sufficiency.
