# Supplementary material for: Comparative functional analyses of PHR1, PHL1, and PHL4 transcription factors in regulating Arabidopsis responses to phosphate starvation
Source: Front Plant Sci. 2024 Apr 19;15:1379562. doi: 10.3389/fpls.2024.1379562 (PMC11066281; doi:10.3389/fpls.2024.1379562)
Supplement: Supplementary Figure 1 — The anthocyanin contents of 12-day-old WT and mutants on +Pi and –Pi media. [file DataSheet_1.pdf]

Supplementary Figure 1

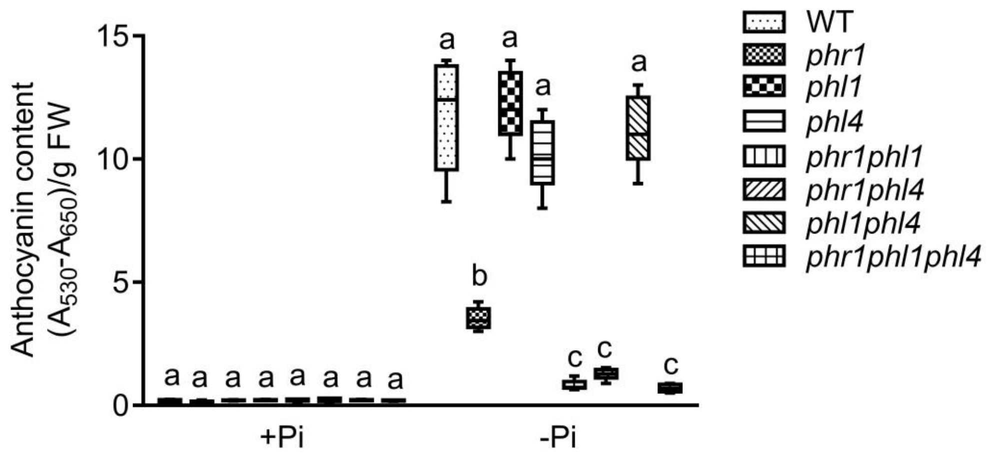

Supplementary Figure 1. The anthocyanin contents of 12-day-old WT and mutants on +Pi and –Pi media. These experiments were repeated three times with similar results. Values represent means ± SD of more than five replicates. Different letters above the columns indicate that these values are significantly different (One-way ANOVA/Tukey, $P < 0.05$).

Supplementary Figure 2

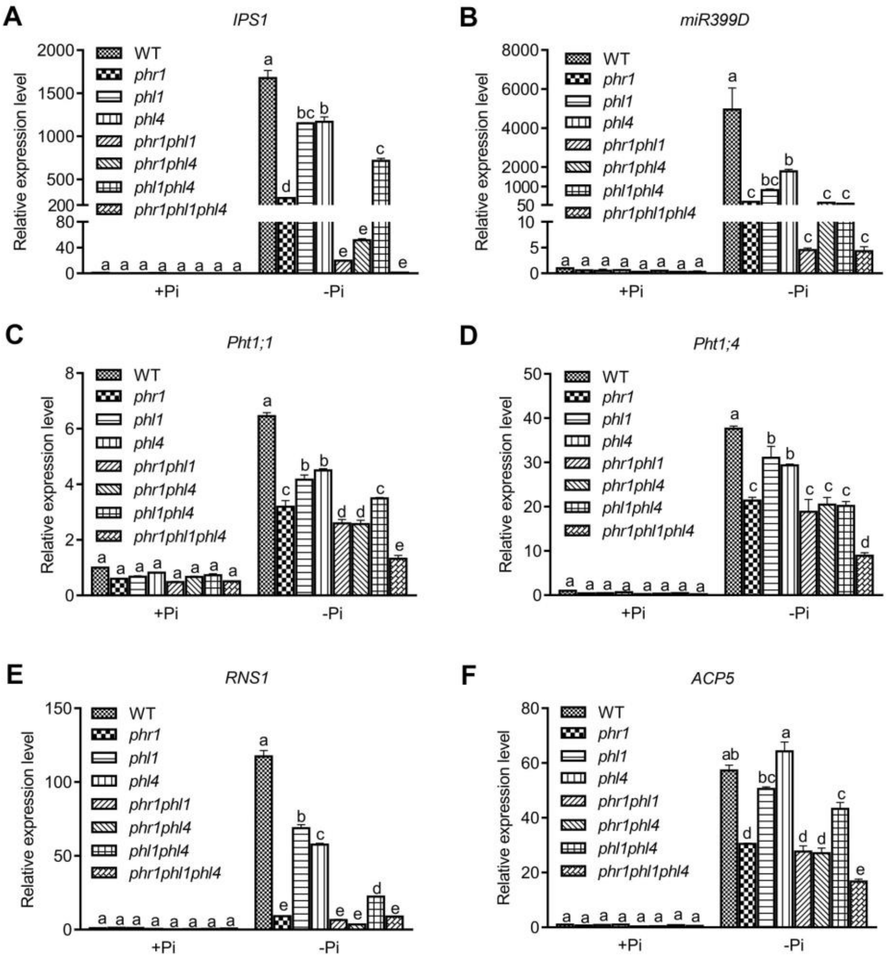

Supplementary Figure 2. Relative expression of six PSI genes in root of the WT and various mutants. Total RNAs were extracted from root of 8-day-old seedlings grown on +Pi and –Pi media. These experiments were repeated three times with similar results. Values represent means ± SD of three replicates. Different letters above the columns indicate that these values are significantly different (One-way ANOVA/Tukey, $P < 0.05$). The names of the genes examined are indicated at the top of each panel.

Supplementary Figure 3

A

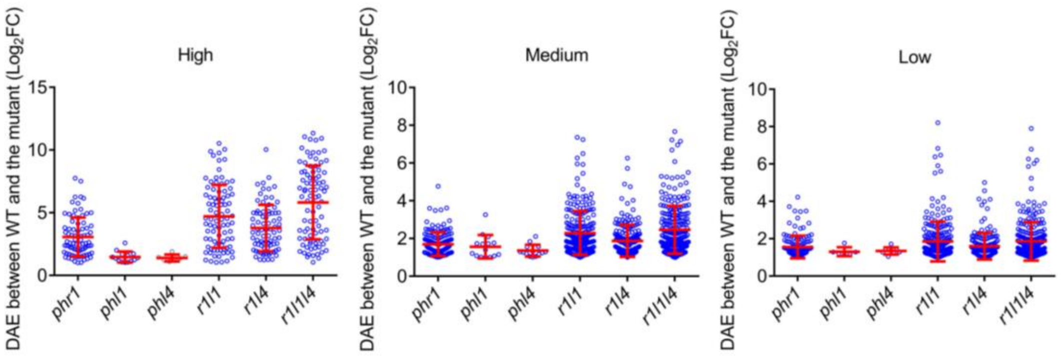

B

Pi Starvation-Responsiveness	DAE between WT and the mutant (Log_2FC)					
	phr1	*phl1*	*phl4*	*r1l1*	*r1l4*	*r1l1l4*
High	3.07	1.47	1.39	4.71	3.78	5.81
Medium	1.70	1.57	1.36	2.29	1.87	2.46
Low	1.59	1.41	1.29	1.87	1.63	1.88

Supplementary Figure 3. The difference of average expression levels (DAE) of mutation-affected PSI genes (PSI-up and PSI-down genes included) between WT and the mutants. (A) The DAE of mutation-affected PSI genes between the WT and each mutant. The scatter plots showing means ± SD. (B) The corresponding data showing in (A). PSI genes were divided into three categories according to their responsiveness to Pi starvation, which "High" refers to $Log_2FC > 5$, "Medium" refers to $2 < Log_2FC \leq 5$, "Low" refers to $1 \leq Log_2FC \leq 2$.
